# Poly(A)-DG: A deep-learning-based domain generalization method to identify cross-species Poly(A) signal without prior knowledge from target species

**DOI:** 10.1371/journal.pcbi.1008297

**Published:** 2020-11-05

**Authors:** Yumin Zheng, Haohan Wang, Yang Zhang, Xin Gao, Eric P. Xing, Min Xu

**Affiliations:** 1 School of Electronic Engineering and Computer Science, Queen Mary University of London, London, United Kingdom; 2 Language Technologies Institute, School of Computer Science, Carnegie Mellon University, Pittsburgh, PA, USA; 3 Computational Biology Department, School of Computer Science, Carnegie Mellon University, Pittsburgh, PA, USA; 4 Computational Bioscience Research Center (CBRC), Computer, Electrical and Mathematical Sciences and Engineering (CEMSE) Division, King Abdullah University of Science and Technology, Thuwal, Saudi Arabia; 5 Machine Learning Department, School of Computer Science, Carnegie Mellon University, Pittsburgh, PA, USA; University of Toronto, CANADA

## Abstract

In eukaryotes, polyadenylation (poly(A)) is an essential process during mRNA maturation. Identifying the *cis*-determinants of poly(A) signal (PAS) on the DNA sequence is the key to understand the mechanism of translation regulation and mRNA metabolism. Although machine learning methods were widely used in computationally identifying PAS, the need for tremendous amounts of annotation data hinder applications of existing methods in species without experimental data on PAS. Therefore, cross-species PAS identification, which enables the possibility to predict PAS from untrained species, naturally becomes a promising direction. In our works, we propose a novel deep learning method named Poly(A)-DG for cross-species PAS identification. Poly(A)-DG consists of a Convolution Neural Network-Multilayer Perceptron (CNN-MLP) network and a domain generalization technique. It learns PAS patterns from the training species and identifies PAS in target species without re-training. To test our method, we use four species and build cross-species training sets with two of them and evaluate the performance of the remaining ones. Moreover, we test our method against insufficient data and imbalanced data issues and demonstrate that Poly(A)-DG not only outperforms state-of-the-art methods but also maintains relatively high accuracy when it comes to a smaller or imbalanced training set.

## Author summary

The key to understanding the mechanism of translation regulation and mRNA metabolism is to identify the *cis*-determinants of PAS on the DNA sequence. PAS leads to correct identification of Poly(A) sites which play an essential role in understanding human diseases. While many researchers have employed deep learning methods to improve the performance of PAS identification, an underlying problem is the expensive and time-consuming nature of PAS data collection, which makes the application of deep learning models for identifying PAS from a broad range of species a tough task. We attempt to use domain generalization methods, inspired by its thrive in the field of computer vision, to overcome the insufficient annotation data challenge in PAS data. Here, empirical results suggest that our proposed model Poly(A)-DG can extract species-invariant features from multiple training species and be directly applied to the target species without fine-tuning. Furthermore, Poly(A)-DG is a promising practical tool for PAS identification with its stable performance on insufficient or species-imbalanced training data. We share the implementation of our proposed model on the GitHub. (https://github.com/Szym29/PolyADG).

This is a *PLOS Computational Biology* Methods paper.

## Introduction

Most eukaryotic mRNA, after being transcribed from its coding DNA, typically undergoes post-transcriptional modification, such as 5’ capping, splicing, and polyadenylation, before it is translocated to the cytoplasm and translated into proteins [1]. Polyadenylation is one of the post-transcriptional modifications, where the 3’ end of a primary transcript mRNA (pre-mRNA) is cleaved and 250-300 adenines are added [2]. Its proposed functions include conferring mRNA stability, promoting mRNA’s translational efficiency, and facilitating the transportation of mature mRNA from the nucleus to the cytoplasm [3–5]. It has been shown that at least two primary *cis*-regulatory elements are needed for polyadenylation: i) a highly conserved AAUAAA hexamer (or a close variant) located at 10-40 nucleotides (nt) upstream of the poly(A) site, which is usually referred to as the polyadenylation signal (PAS) [6, 7] ii) a poorly conserved GU- or U-rich RNA sequence located at 20-40 nt downstream of the polyadenylation site [8, 9].

The location of the poly(A) site determines the length of the 3’ untranslated regions (3’-UTR), which further modulates the expression and of the gene [10, 11]. Recent studies revealed that more than 70% of eukaryotic genes have more than one polyadenylation site and produce multiple RNA isoforms through the usage of different cleavage of poly(A) sites, a process named alternative polyadenylation [12–15]. These RNA isoforms have the same coding regions but differ in their 3’ UTRs. The differed 3’ UTRs may confer different stability, translation efficiency, functions or subcellular localization to the mRNA isoform [16]. Importantly, it has been implicated in a wide range of human diseases, including cancer and neuromuscular disorders [2, 17]. A single nucleotide change of the PAS could lead to dysregulation of *TP*_53_ expression and has been associated with the progression of thyroid tumors. [18, 19] Therefore, correct identification of poly(A) sites plays a crucial role in understanding diseases mechanisms. It is well known that the PAS determines the correct identification of poly(A) sites [7, 20] and their mutations directly associated with diseases. Therefore, studies on PASs and their surrounding regions are significant for biological researches. Recently, technologies combining biochemistry and high-throughput sequencing such as DRS, PAS-seq [21], 3P-seq [22], SAPAS [23], 3’READ [24], and RNA-seq [25] have been developed to identify genome-wide poly(A) signal with a high accuracy. However, it is known that polyadenylation is tissue-specific, so experiments are required for each tissue. Although experimental methods provide promising results, researchers began to seek help for computational methods to accurately and conveniently identify PAS.

Computational methods enable efficient PASs identification by learning the contexts of PASs motif and discriminating true PASs related DNA sequences from sequences which don’t contain PAS. Ji et al. [1] modified a Generalized Hidden Markov Model (GHMM) to extend the utility of the Hidden Markov Model (HMM) by giving each state multiple observed values such that it can easily be used in describing the organization of gene sequences. With the demonstrated strong empirical performance of the support vector machine (SVM), researchers began to introduced SVM based models in PAS identification. [26, 27] More recently, HSVM [28], derived a set of latent features by HMM and fed features to a SVM for classification, increased the accuracy of poly(A) recognition on a public benchmark, Dragon human dataset [29]. Omni-PolyA [30] further boosted the accuracy of PAS identification by combining multiple classification models, including decision tree, RF, etc. However, even these traditional machine learning models are competent to identify PAS, they have a common drawback that is they are built for specific hand crafted features which are costly and time-consuming to obtain. Therefore, researchers began to seek more general methods to automatically identify PAS from DNA sequences.

Deep learning methods have been proposed to automatically learn features of DNA sequences and predict unknown sequences based on learned knowledge. With the increasing popularity of Convolution Neural Network (CNN), DeepPolyA [31] first applied CNN in PAS identification. They proposed a two-convolution-layer model and showed that their model outperformed many other deep learning models in PAS identification in plant *Arabidopsis thaliana* coding DNA. In 2019, Xia et al. [32] proposed DeeReCT-PolyA, a shallow CNN model mainly consists of one convolution layer, two fully connected layers, and exhibited higher performances compared with previous deep learning models on four public PAS dataset. Later, Kalkatawi et al. [33] built DeepGSR to recognize different types genomic signals and regions, including PAS, in genomic DNA. Recently, Yu et al. [34] developed a self-attention deep learning model and outperforms the state-of-the-art PAS identification methods. However, the datasets availability for training neural networks are often limited in scale; thus it limits the performance of the model. Although Xia et al. [32] extended their pre-train models for a new species or new PAS variants to mitigate the paucity of annotated data, their models required a large amount of annotated sequences from the target species, which hindered their method in practice. DNA sequences from different species may have different gene regulations and functional meanings. We thereby assume that PAS data from different species have different sequence preference (distributions). Traditional computational methods hardly adapt to different species since they are designed to learn hand-crafted features for specific distributions. Domain adaptation (DA), an important field of deep learning, describes how to apply a well-trained deep learning model in a different (but related) target data distribution to address same tasks. Domain generalization (DG), as a variant of DA, further improves the generalization of a model for requiring no prior knowledge of target data distribution.

DeeReCT-PolyA [32] first provided clues to apply DA in addressing the issue of lack of annotated PAS data in some species. In this work, we take a further step to seek a DG method to identify PAS in some species without any experimental data. We focus on improving the generality of deep-learning model by learning PAS regulation features shared by different species [35] and identifying PAS from some species without prior knowledge.

The main contributions of this paper are organized as follows:

- We propose a neural network CNN-MLP to extract both motif features and species information about PAS related DNA sequences.
- We first introduce a domain generalization technique, HEX, in PAS recognition to force the model to learn invariant features so that the model can generalize to different species.
- We evaluate Poly(A)-DG by learning from a cross-species dataset and predicting PAS of new species directly.
- We investigate the performance of Poly(A)-DG when there are limited amount of training data or they are imbalanced.
- We explore the performance of Poly(A)-DG in imbalanced ratio of positive and negative PAS DNA samples.
- We visualize the motif patterns captured by convolution filters, and the contribution of regions of DNA sequence.
- We evaluate the performance of Poly(A)-DG by predicting whole genome in chromosome 1 of rat.
- We explore the homology effect of Poly(A)-DG in cross-species PAS identification.
- We also investigate how the Poly(A)-DG performs on single PAS genes and multiple PAS genes.

## Methods

The main challenge of identifying PAS of new species is the limitation of annotated data [32, 36], especially in the case when we have no annotated data at all. We cannot directly apply well-trained poly(A) signal identification models to a new species for two reasons. First, patterns and locations of poly(A) signals vary widely in different species [37]. Moreover, Xing et al. [38] speculates that there are subtle differences of polyadenylation signal recognitions or uncharacterized species-specific polyadenylation enhancers leading to differential processing. Fortunately, however, the in-depth DNA sequences connection among different species can be employed to identify PAS for widespread species. Therefore, we propose our method, Poly(A)-DG. We will first introduce our features extraction module, CNN-MLP that captures both motif features and general sequence information; then we will introduce a technique to remove species-specific information from motif features such that the model is able to learn the invariant motif features among different species to identify PAS of a new species without experimental data.

### Data description

The data sample, *j*, denotes as < **X**_*j*_, **Y**_*j*_ > where **X**_*j*_ is a DNA sequence and **Y**_*j*_ is the annotation. The length of each DNA sequences is 206-nt and PAS locates in the middle of sequences where its upstream and downstream are both 100-nt. DNA sequences need to be one-hot encoded into four-channel data sequences and each channel represents a type of nucleobase. The annotation of each DNA sequence is {0, 1}, denoting whether the sequence contains PAS or not.

### CNN-MLP for feature extraction

We propose a neural network aiming to automatically extract both general features and motif features of DNA sequences from different species.

Multilayer Perceptron (MLP) has a good reputation for portraying the whole distribution owing to each node in the hidden layers connected with a certain weight to every node in previous layers. Each node in the hidden layer can be considered as a sequence descriptor that scans through all nucleotides of inputs DNA sequences and obtain a score to describe the sequence. In the training session, MLP tends to fit the training data and try to find proper weights vectors to describe the training set accurately. Given a DNA sequence dataset consists of different species, the MLP would learn to describe the general information among species instead of learning to fit specific features. Therefore, we try to employ the MLP to extract general information about different species. Previous work [32] showed that the simpler architecture would lead to higher performance; thus we construct our MLP with only one hidden layer and use ReLU to activate it. The representation of MLP denotes by *F*_*G*_ (Eq (1)) where *X* is a set of raw data fed in the MLP, *σ* is activation function (ReLU in our model), *W*_*i*_ is a vector of weights that hidden nodes used to connect with nodes in the input layer and *b*_*i*_ is a set of bias.

$$\begin{array}{c} F_{G}=\sigma \left(W_{i}\cdot X+b_{i}\right) \end{array}$$ (1)

Different species share the same PAS patterns and flanking motifs may relate to PAS regulations. For using MLP to extract features that can describe species instead of PAS related motifs, we use shuffled-DNA as the inputs of MLP. Therefore, the representations, namely confounding species-specific factors, extracted by MLP intend to describe the specific species and become impossible to identify PAS. The comparison of performance of feeding shuffled DNA sequences and non-shuffled DNA sequences to MLP is shown in section 2 of S1 Supporting Information.

Then we construct another neural network aiming to identify PAS in single species and use domain generalization techniques to enable cross-species PAS identification by neutralizing the influence of species-specific features extracted by MLP. We feed non-shuffled DNA sequences into a Convolution Neural Network (CNN), which is famous for extracting motif features from DNA sequences. As shown by previous works [31, 32], CNN can be applied to extract the motif information from DNA sequences to identify PAS. Compared with MLP, CNN directly deals with original DNA sequences instead of shuffling them so that the motif structure of DNA sequences can be retained. In order to keep the architecture simpler for better performance [32] we build a CNN only contain a single convolution layer that consists of several convolution kernels. Each kernel serves as a motif detector [32], and it is able to capture sequence motifs, which are short, recurring patterns in DNA sequences that are presumed to have a biological function. In the training process when inputs are fed to the convolution layer, convolutional kernels can search neighboring bases for their relationship and automatically infer the most relevant motif features [31]. ReLU activates the output of the convolution layer and sends motif features to a max-pooling layer so that the number of parameters could be reduced. Dropout technique [39] is used to prevent the network from sticking at a local optimum and assuage over-fitting.

**Fig 1:**
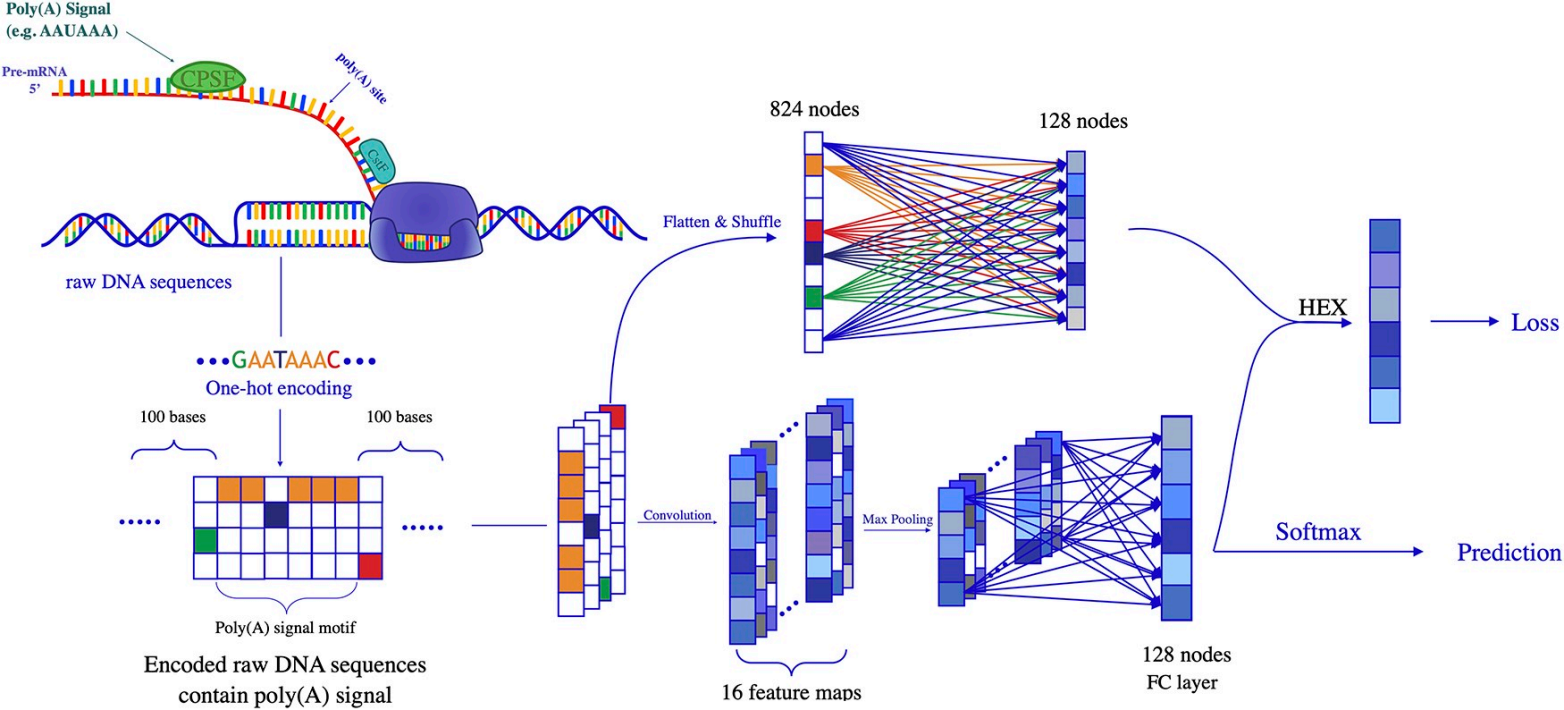
This figure illustrates the architecture of Poly(A)-DG. Each pair of inputs includes a raw DNA sequence and its label to show it contains PAS or not. The raw DNA sequences are encoded into the one-hot format and send into CNN and MLP sub-network. The outputs of CNN and MLP are concatenated and used HEX technique to minimize the differences among different species. The output of CNN is directly used in prediction after softmax and the output of HEX is used in loss calculation. The back-propagation algorithm is employed to tune the model with loss.

Fig 1 shows our feature extraction method, raw DNA sequences are encoded into the one-hot format and then fed into the neural network. MLP flattens the shuffled-DNA data and connects input nodes with weight *W*_*i*_ to each hidden node. The inputs are directly fed to CNN and 16 convolution kernels are used to capture motif features. The architecture of feature extraction is detailed section in 1.1 & 1.2 in S1 Supporting Information.

### Domain generalization technique

As the variance of the distributions may not be simply coded in some available labels, invariance-driven methods such as DANN [40] and other similar methods [41–43] will not be applicable. Therefore, we adopt a recently proposed unguided domain generalization technique, HEX [44], aimed to force the model to focus on invariant features and doesn’t need the labels of distributions. We use HEX to force the model to ignore the species-specific upstream and downstream context information and focus on bases that contributed to PAS identification. As we show in Fig 1, the output of CNN sub-network, *F*_*A*_ is directly used in predicting whether the sequence contains PASs or not. The outputs of CNN sub-network and MLP sub-network are concatenated and then fed into HEX. Now we need to force the model to learn species-invariant features. With *F*_*A*_ and *F*_*G*_ used in Eq 2, we need to transform the representation of *F*_*A*_ so that it is least explainable by *F*_*G*_. Directly adopting subtraction maybe problematic because the *F*_*A*_ − *F*_*G*_ can still be correlated with *F*_*G*_. Wang et al. [44] proposed a straightforward way is to regress the information of *F*_*G*_ out of *F*_*A*_. Since both *F*_*A*_ and *F*_*G*_ are in the same space and the only operation left in the network is the argmax operation, which is linear, we can safely use linear operations.

To form a standard linear regression problem, we first consider the column *k* of *F*_*A*_, denoted by $F_{A}^{\left(k\right)}$. To solve a standard linear regression problem is to solve:

$$\begin{array}{c} \hat{\beta^{\left(k\right)}}=\underset{\beta^{\left(k\right)}}{\arg\min}\left|\right|F_{A}^{\left(k\right)}-F_{G}\beta^{\left(k\right)}{\left|\right|}_{2}^{2} \end{array}$$ (2)

*β* is the coefficient vector of linear regression in Eq 2. This function has a closed form solution $\hat{\beta^{k}}$ when the mini-batch size is greater than the number of classes of the problem (*i.e*. when the number of rows of *F*_*G*_ is greater than number of columns of *F*_*G*_), and the closed form solution is:

$$\begin{array}{c} \hat{\beta^{\left(k\right)}}=\frac{F_{G}^{T}F_{A}^{\left(k\right)}}{\left(F_{G}^{T}F_{G}\right)} \end{array}$$ (3)

Therefore, for *k*^th^ column of *F*_*A*_, what cannot be explained by *F*_*G*_ is (denoted by $F_{L}^{\left(k\right)}$):

$$\begin{array}{c} F_{L}^{\left(k\right)}=F_{A}^{\left(k\right)}-F_{G}\frac{F_{G}^{T}F_{A}^{\left(k\right)}}{\left(F_{G}^{T}F_{G}\right)}=\left(I-F_{G}{\left(F_{G}^{T}F_{G}\right)}^{-1}F_{G}^{T}\right)F_{A}^{\left(k\right)} \end{array}$$ (4)

Repeat this for every column of *F*_*A*_ will lead to:

$$\begin{array}{c} F_{L}=\left(I-F_{G}{\left(F_{G}^{T}F_{G}\right)}^{-1}F_{G}^{T}\right)F_{A} \end{array}$$ (5)

which is how HEX works to focus on the invariant features of cross-domain datasets and naturally generalizes to a new species not used in training. Then we use the *F*_*L*_ for parameters tuning.

We employ Binary Cross Entropy (BCE) as the cost function to calculate the loss of every training epoch. Adam optimizer [45] is used to minimize the loss derived by BCE. In order to make the training process more stable, we apply an exponential decay method to decrease the learning rate every 1000 iterations. The domain generalization and classification module is detailed in section 1.3 in S1 Supporting Information.

### Cross-species training and hyper-parameter search

Inspired by the cross-validation strategy, we employ a new cross-species training strategy in our work. We randomly divide every training species into five folds and define three of them as training fold and the other two folds as optimization fold. The training fold is used in model training and the optimization fold is used in searching optimal models. The accuracy of cross-species training is obtained by testing models on all sequences from the target species.

The random sampling strategy is applied to search a relative better hyper-parameter for the different training sets. We evaluate those hyper-parameter sets in optimization folds and select the one with the best performance as the parameter of the final model. In our experiments, this method helps us choose hyper-parameters such as learning rate and keep probability. Details can be found in section 1.4 in S1 Supporting Information.

## Results

### Datasets

We use PAS datasets from four species, Mouse, Rat, bovine, and Human, in our experiments. The Omni Human Poly(A) dataset is recently established by Magana-Mora et al. [30] which contains 37,572 sequences and the number of true PAS sequences equals the number of pseudo-PAS ones. The human pseudo-PAS sequences are produced by excluding all the true PAS sequences derived from human chromosome 21. C57BL/6J (BL) Mouse PAS dataset, which has 13 PAS motif variants, consist of 46,224 sequences, is produced by Xia et al. [32]. The bovine dataset were established by Kalkatawi et al. [33] which contains 12,082 true PAS sequences and the they extracted false PAS sequences from chromosome 28 for bovine which also contains 12,082 DNA sequences.According to a Rat Poly(A) database [46], we extract the annotated PAS DNA sequences from genome database to build a Rat true-PAS dataset. Rat true-PAS dataset can be found in S2 File. Rat pseudo-PAS dataset is established by scanning the genomic sequences of the transcripts expressed in the same cell lines and selecting those that are not close to any annotated transcription end in GENCODE or any poly(A) sites identified by the experimental data. Rat pseudo-PAS dataset can be found in S3 File. Our Rat Poly(A) dataset has the same PAS motif variants with human and it is composed of 42,008 sequences. The specific procedure can be found in section 3 in S1 Supporting Information.

PAS patterns have various variants, we have thirteen PAS motif variants from mouse and twelve from human and Rat poly(A) dataset. However, in this paper, we only focus on identifying the same PAS variants among different species; thus we select the eleven PAS motif variants that Omni human, Rat, bovine and BL mouse shared. Therefore, we use remained 37,230 sequences from Omni human Poly(A), 42,428 for BL mouse, 42,008 for rat and 22,782 for bovine in our experiments. Human, bovine and Mouse PAS datasets can be found in S1 File. Each poly(A) dataset is composed of the same number of positive and pseudo-PAS sequences.

### Comparison methods

We compare Poly(A)-DG with our feature extraction module, MLP-CNN, to evaluate whether HEX can force the model to learn invariant features among different species or not, DeeReCT-PolyA (DeeReCT), [32] which is a state-of-the-art domain adaptation method in PAS identification and state-of-the-art deep learning methods in PAS identification including DeepPolyA [31] and SanPolyA [34]. Although DeepGSR [33] can identify PAS with a high accuracy, it doesn’t fit the data used in our work. We train Poly(A)-DG, MLP-CNN, DeepPolyA and SanPolyA on the cross-species datasets and predict the PASs in the remaining species that not used in training. DeeReCT-PolyA applies a fine-tune strategy, it first trains on a source species then fine-tunes the model on a target species and then test on new species.

### Standard experiment

Owing to different distributions of DNA sequences among different species, people can hardly use a well-trained model to identify PAS of a new species. Therefore, we want to evaluate whether Poly(A)-DG is promising to learn invariant PASs related features from a cross-species dataset to overcome domain shifts or not.

First, we build six cross-species training sets, including Human-Mouse, Human-Rat, Mouse-Rat, Rat-bovine, Mouse-bovine, and Human-bovine, by mixing any two datasets discussed in Section Datasets and define the remaining species as the target species. Following the cross-species training method in Section Cross-species training and hyper-parameter search, we split each species in cross-species training sets into the training fold and the optimization fold. Then we train models on training folds and report the average accuracy over the target species. In experiments, we observe that the best learning rate and dropout rate vary when the models train on different cross-species datasets. Therefore, we run every methods a few times to obtain their best performance on different source domains. Experimental results are shown in Tables 1, 2, 3, 4, 5 and 6.

**Table 1:** Source domain: Omni human and BL mouse. Target domain: Rat and bovine.

methods	Rat	bovine
Poly(A)-DG	**68.5%**	75.3%
CNN-MLP	65.4%	74.2%
SanPolyA	68.4%	**75.6%**
DeepPolyA	68.1%	73.7%
DeeReCT fine-tune on BL mouse	65.9%	70.8%
DeeReCT fine-tune on Human	65.4%	73.7%

**Table 2:** Source domain: Human and bovine. Target domain: BL mouse and rat.

methods	Mouse	Rat
Poly(A)-DG	**68.9%**	**69.4%**
CNN-MLP	67.7%	65.1%
SanPolyA	67.5%	61.6%
DeepPolyA	66.2%	67.4%
DeeReCT fine-tune on Human	67.8%	65.3%
DeeReCT fine-tune on bovine	67.4%	64.6%

**Table 3:** Source domain: BL mouse and bovine. Target domain: Human and rat.

methods	Human	Rat
Poly(A)-DG	**75.0%**	**67.6%**
CNN-MLP	73.6%	64.3%
SanPolyA	74.3%	63.7%
DeepPolyA	72.1%	66.5%
DeeReCT fine-tune on Mouse	70.3%	66.0%
DeeReCT fine-tune on bovine	74.1%	65.3%

**Table 4:** Source domain: Omni human and rat. Target domain: BL mouse and bovine.

methods	Mouse	bovine
Poly(A)-DG	**69.7%**	**76.0%**
CNN-MLP	68.3%	75.3%
SanPolyA	69.4%	75.7%
DeepPolyA	67.8%	74.3%
DeeReCT fine-tune on Rat	66.5%	69.5%
DeeReCT fine-tune on human	68.5%	74.3%

**Table 5:** Source domain: Rat and BL mouse. Target domain: Omni human and bovine.

methods	Human	bovine
Poly(A)-DG	**72.3%**	**72.4%**
CNN-MLP	70.7%	70.7%
SanPolyA	72.1%	72.1%
DeepPolyA	70.6%	70.7%
DeeReCT fine-tune on Rat	68.6%	67.6%
DeeReCT fine-tune on Mouse	71.3%	68.3%

**Table 6:** Source domain: Rat and bovine. Target domain: Human and BL mouse.

methods	Human	Mouse
Poly(A)-DG	73.8%	67.9%
CNN-MLP	73.2%	67.7%
SanPolyA	73.5%	**68.1%**
DeepPolyA	71.0%	66.6%
DeeReCT fine-tune on Rat	68.3%	66.2%
DeeReCT fine-tune on bovine	**74.3%**	66.8%

Generally speaking, Poly(A)-DG outperforms remaining methods. Compared with CNN-MLP, Poly(A)-DG achieves higher accuracy in all experiments in this section by employing HEX module. For example, in Table 1, the accuracy of Poly(A)-DG is 3.1% higher than CNN-MLP for Rat and 1.1% higher for bovine. Similar conclusions can be drawn from remaining tables. We observed that HEX improves empirical score, and we conjecture that HEX successfully ignores some influences of domain specific features and forces the model to learn invariant features which are essential for cross-species PAS identification. Moreover, Poly(A)-DG achieves better and stable performances when it compared with state-of-the-art deep learning models. As we mentioned in Section Comparison methods, SanPolyA and DeepPolyA take the same training strategy as Poly(A)-DG. From empirical results in this section, we can directly find that Poly(A)-DG is better than DeepPolyA. In general, SanPolyA performs as good as Poly(A)-DG, but in some scenarios, the accuracy for Rat in Tables 2 and 3, SanPolyA can not achieve a relatively high accuracy. Compared with SanPolyA, the performance of Poly(A)-DG is more stable in all groups of experiments. The accuracy of DeeReCT is influenced by the order of species used in pre-train and fine-tune. If we use an improper training order, the performance of DeeReCT would be mediocre while the appropriate training order would improve the performance of the model. In Table 4, the accuracy of Poly(A)-DG is 3.2% and 6.2% higher than DeeReCT when it pre-trains on Human and fine tunes on Rat. When the order of pre-train and fine-tune is reversed, the results of DeeReCT increase to 68.5% and 74.3%, which are closer to the ones of Poly(A)-DG.

Compared with DeeReCT, we notice that Poly(A)-DG does not need to find out the most suitable pre-train and fine-tune order to obtain the highest testing accuracy. We conjecture that Poly(A)-DG can simultaneously learn the features from all species in the source domain and reduce the superficial species-specific information automatically. Due to problems in the order of pre-train and fine-tune of DeeReCT and reliability of SanPolyA, Poly(A)-DG is more promising in resolving practical tasks.

### Training with limited data

In addition, we want to investigate the effectiveness of Poly(A)-DG given different amounts of data, especially when the amount of data is limited. Therefore, we define the training fold used in the Section Standard experiments as the standard training fold and further uniformly split each standard training fold into ten sub-folds. The size of a new training fold ranges from 10% to 100% of the standard training fold by increasing sub-folds. We show eight groups of experiments in this section and other four groups of experiments can be found in the section 4 in S1 Supporting Information.

**Fig 2:**
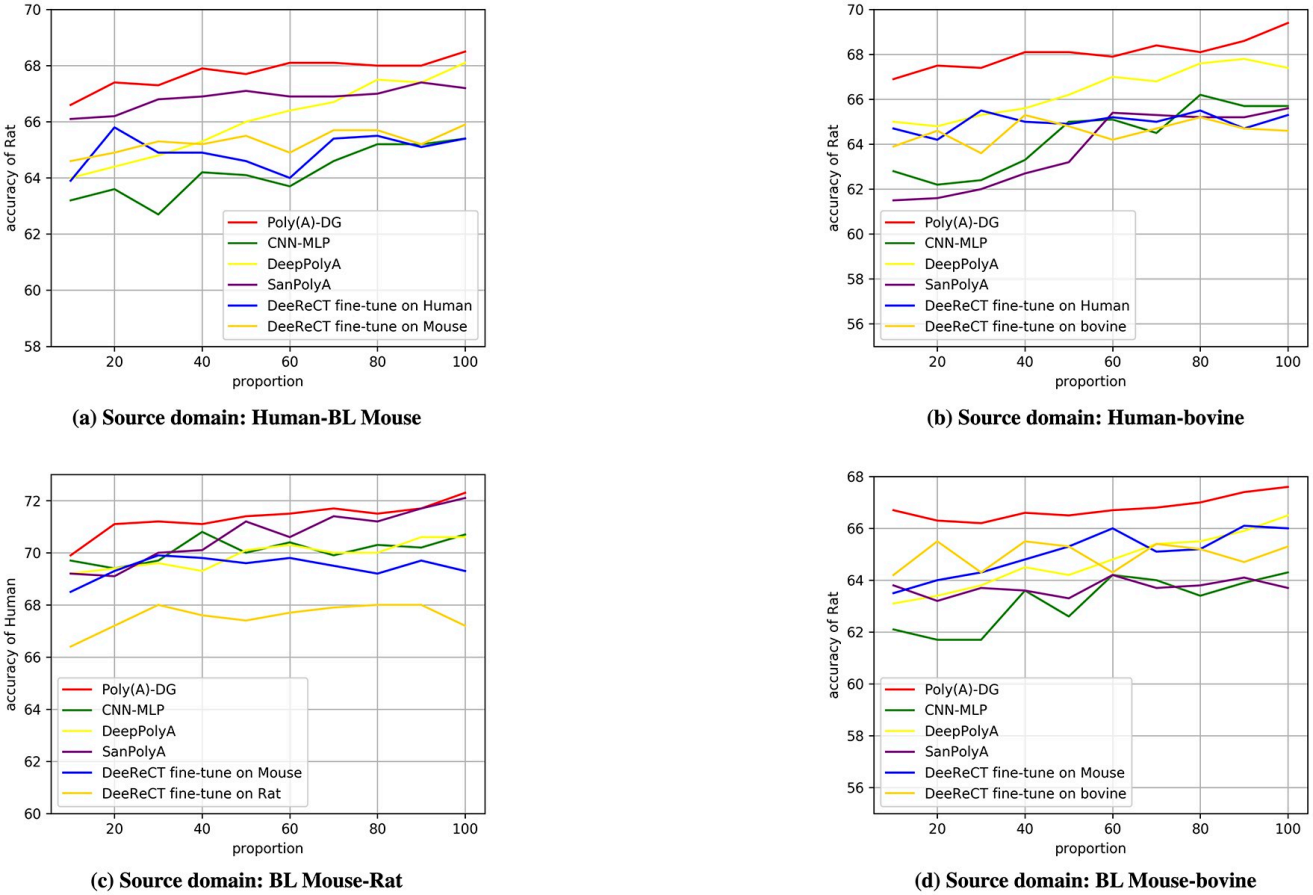
The data from different species are in the same scale. (a)training source domain is mixed by Omni Human and Mouse, target species is Rat. (b) training source domain is mixed by Omni Human and bovine, target species is Rat. (c) training source domain is mixed by Rat and Mouse, target species is Omni Human. (d) training source domain is mixed by bovine and Mouse, target species is Rat.

In general, the accuracy of Poly(A)-DG has an increasing tendency when the amounts of sequences are increasing. Impressively, in Fig 2b and 2d when the number of sequences used in training is only one-tenth of the standard training fold, the accuracy of Poly(A)-DG is still higher than other methods even when the full training fold is available. From these eight sub-figs, we observe that Poly(A)-DG always performs better than CNN-MLP and DeepPolyA. The performance of DeeReCT depends on the pre-train and fine-tune order. The performance of DeeReCT would be undesirable if it takes a wrong pre-train and fine-tune order, but DeeReCT pre-trained on Rat is competitive with Poly(A)-DG. When the data is limited, the accuracy of DeeReCT pre-trained on Rat is higher than Poly(A)-DG. While the number of sequences used in training increases, Poly(A)-DG performs better than DeeReCT. SanPolyA is the strongest competitor of Poly(A)-DG among comparison methods, but it performs less steadily than Poly(A)-DG. As we discussed above, HEX is speculated to force the model to learn invariant features among different distributions. Therefore, Poly(A)-DG may not work effectively over similar data distributions. The sequences come from different tissues of species; some tissues provide significant more data than other tissues. Also, we have 11 poly(A) motifs, the number of some motifs is sufficient while the number of other motifs is limited. When we shuffle the dataset and pick up sequences randomly in training, sequences are more likely to come from those tissues or motifs contained more sequences and we conjecture that these sequences between Mouse and Rat or Rat and Human have a higher similarity. When the training set becomes larger, the training set contains more diverse sequences, which may decrease the similarity between different species, so Poly(A)-DG is likely to work.

Our experiments demonstrate that Poly(A)-DG can be applied in addressing the insufficient data problems.

### Training with imbalanced species

**Fig 3:**
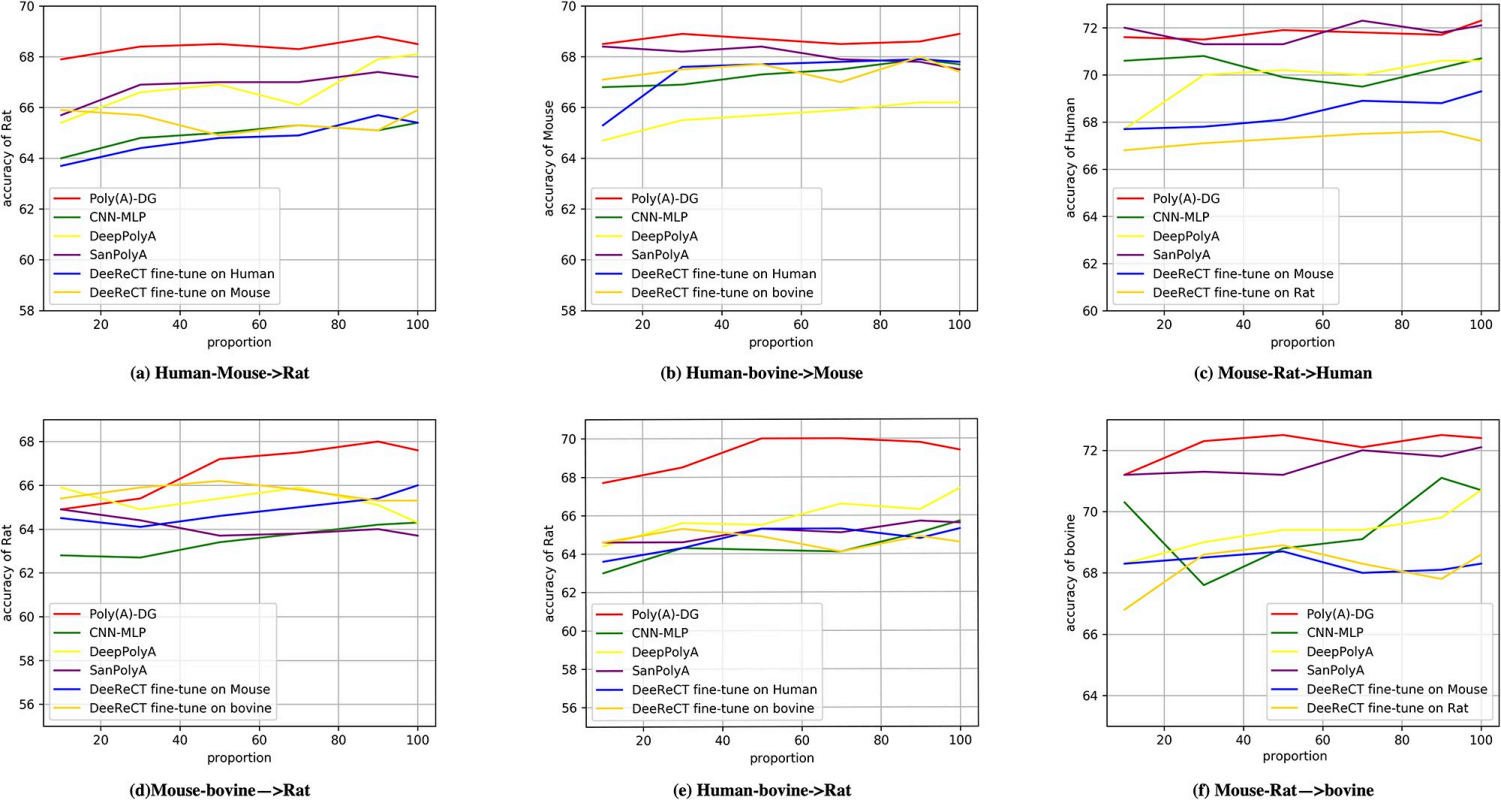
Imbalanced source domains: The number of data from the first source species is fixed. (a)source domain: Omni Human and Mouse, target species: Rat. (b) source domain: Omni Human and bovine, target species: BL Mouse. (c) source domain: BL Mouse and Rat, target species: Omni Human. (d) source domain: BL Mouse and bovine, target species: Rat. (e) source domain: Omni Human and bovine, target species: Rat. (f) source domain: BL Mouse and Rat, target species: bovine.

**Fig 4:**
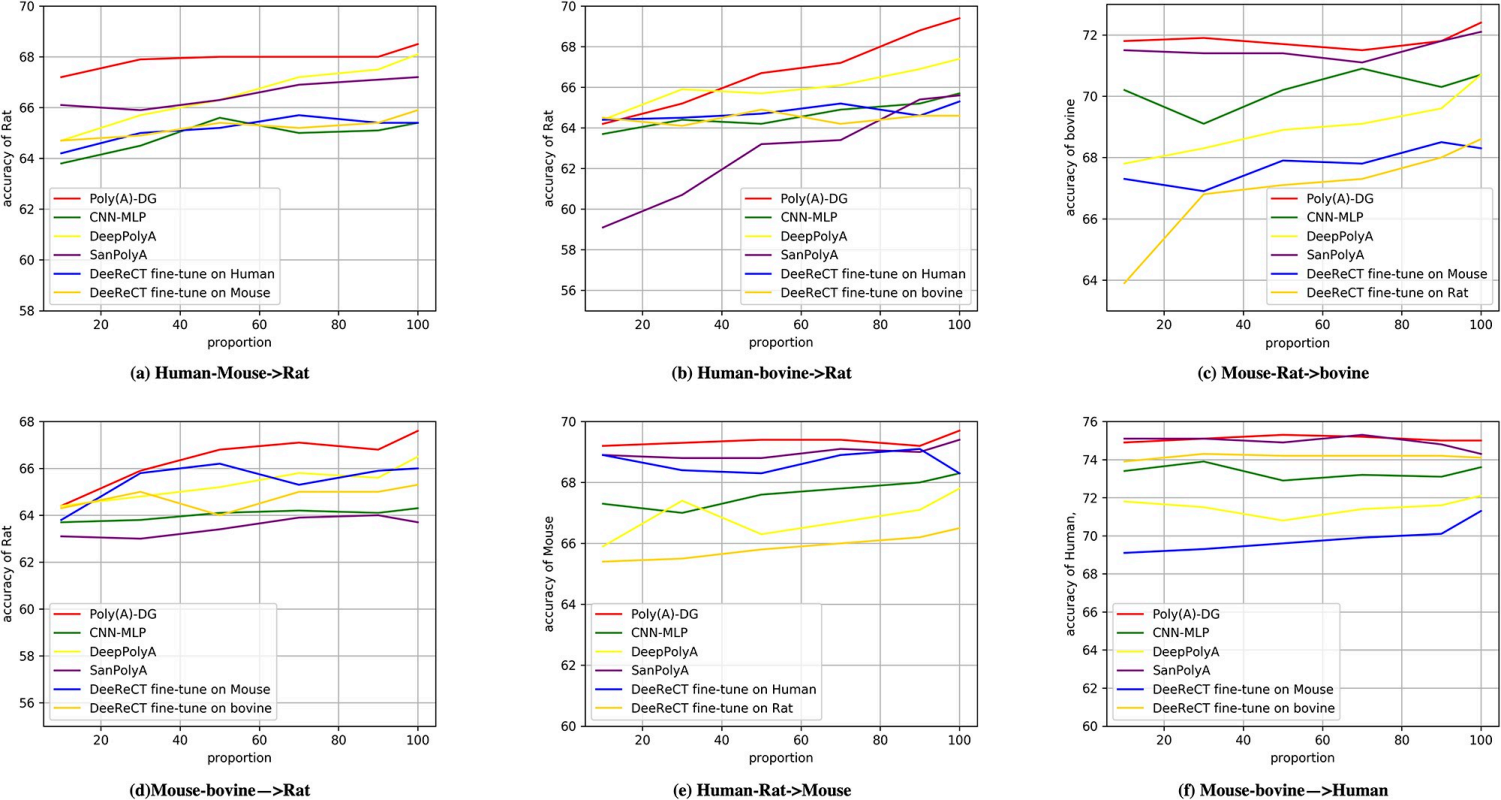
Imbalanced source domains: The number of data from the second source species is fixed. (a)source domain: Omni Human and Mouse, target species: Rat. (b) source domain: Omni Human and bovine, target species: Rat. (c) source domain: BL Mouse and Rat, target species: bovine. (d) source domain: BL Mouse and bovine, target species: Rat. (e) source domain: Omni Human and Rat, target species: BL Mouse. (f) source domain: BL Mouse and bovine, target species: Omni Human.

We intend to investigate the performance of our method dealing with variations of sample abundance of the species, because the amount of available data for different species may differ, training sets are usually imbalanced to take advantage of the abundance of the data sets. However, insufficient species may become noise in training due to distribution differences and even confuse the model so that it produces undesirable predictions. [47, 48] While we still adopt the data splitting strategy discussed in Section Training with limited data, in this section, we start by training Poly(A)-DG on the standard training fold and gradually decrease the number of sub-folds of one species in the cross-species training set and the number of sequences of the other species remains the same. We have six pairs of source domain, Omni Human-BL Mouse, Omni Human-Rat, Omni Human-bovine, BL Mouse-Rat, BL Mouse-bovine and Rat-bovine. We fix the first species in the source domains mentioned above and plot the empirical results of experiments conducted on them in Fig 3. The results of experiments conducted on source domains that the second species is fixed are plotted in Fig 4. We show experimental results of six groups experiments for Figs 3 and 4 in this section, other twelve group of experiments can be found in section 5 of S1 Supporting Information. We observe that the performance of Poly(A)-DG is obvious better than over methods in some sub-figures such as Figs 3a, 3e and 4a. Not only the accuracy of Poly(A)-DG is higher than others, but the accuracy is more steady when the number of sequences of one species decreases. The performance of SanPolyA is very close to Poly(A)-DG in some pairs of experiments but in some sub-figures, for example, Fig 3d, the accuracy declines when the number of bovine increases in the source domain. In the experiments, we find that in different source domains or in the same source domain with different ratios of the number of sequences for each species, DeepPolyA is very sensitive to the learning rate. Although the accuracy reported in Figs 3 and 4 is acceptable, in worst cases, DeepPolyA cannot even converge.

In some situations, the performance of DeeReCT performs close or even better than Poly(A)-DG, but when it comes to another species in target domains or takes other order of pre-train and fine-tune, the performance will be undesirable.

In contrast, experimental results show that Poly(A)-DG can adapt to different cross-species datasets and it works stably when the size of insufficient species are various. We also find that CNN-MLP does not work as effectively as Poly(A)-DG. We conjecture that, confused by the imbalanced training set, CNN-MLP could mistakenly learn species-specific motif information from cross-species datasets.

The result of experiments shows that Poly(A)-DG can be promising in addressing imbalanced dataset problems.

### Training with imbalanced PAS positive-negative ratio

In experiments conducted above, we assume that the ratio of the number of positive and negative PAS is equal. However, in real conditions, the number of pseudo PAS sequences could be higher than true PAS sequences and the ratio of positive and negative samples may vary among species. In this section, we train Poly(A)-DG in new training sets in different ratios of positive and negative PAS, including 1:5, 1:3 and 1:2 and predict PAS from new species with equal number of false and true PAS sequences. We set the Poly(A)-DG models training on standard training sets as control groups. Experimental results are shown in Table 7. When the ratio of negative and positive PAS is 5:1, though the accuracy of PAS identification decrease by about 10% to 13%, Poly(A)-DG is still able to recognize PAS sequences. The performance of Poly(A)-DG is closer to control groups by having more true PAS sequences. When the ratio of negative and positive PAS becomes 2:1, Poly(A)-DG can even achieve higher performance than control groups. We surmise that Poly(A)-DG may learn enough invariant PAS related features from the 2:1 negative-positive PAS datasets and extra true PAS sequences sometimes cannot provide more species-invariant motif features with Poly(A)-DG but more species-specific information. We also research the performance of Poly(A)-DG training on soruce domains with a ratio between positive and negative is 1:10. (Section 6 in S1 Supporting Information) We further investigate if Poly(A)-DG can work on cross-species datasets that the ratio of positive and negative samples is different for different species. In this group of experiments, we set the ratios of positive and negative PAS sequences are 1:3 and 1:2 for different speices in the source domain. The details can be found in Table 8. These experimental results show that the ratio of the number of positive and negative samples affect the performance of Poly(A)-DG, but the model can still work to identify PAS.

**Table 7:** Imbalanced PAS positive-negative ratio.

Positive: Negative											
Source Domain		Target	1:5	1:3	1:2	1:1	Target	1:5	1:3	1:2	1:1
Omni Human	BL Mouse	Rat	55.5%	59.4%	63.1%	68.5%	bovine	62.2%	68.0%	72.9%	75.3%
Omni Human	Rat	BL Mouse	63.5%	67.8%	70.2%	69.7%	bovine	66.7%	72.3%	75.5%	76.0%
Omni Human	bovine	BL Mouse	62.4%	66.6%	69.1%	68.9%	Rat	65.7%	70.9%	73.3%	69.4%
bovine	BL Mouse	Omni Human	60.6%	66.2%	71.8%	75.0%	Rat	55.3%	58.9%	62.1%	67.6%
Rat	BL Mouse	Omni Human	69.6%	66.5%	70.7%	72.3%	bovine	59.7%	66.6%	70.4%	72.4%
Rat	bovine	Omni Human	63.3%	68.4%	72.5%	73.8%	BL Mouse	62.3%	65.9%	68.7%	67.9%

**Table 8:** Imbalanced PAS positive-negative ratio for different species; 1:2 for the first source species and 1:3 for the second one.

Source Domain		Target	Accuracy	Target	Accuracy
Omni Human	BL Mouse	Rat	58.0%	bovine	66.8%
Omni Human	Rat	BL Mouse	63.7%	bovine	68.1%
Omni Human	bovine	BL Mouse	68.4%	Rat	65.4%
bovine	BL Mouse	Omni Human	63.8%	Rat	57.3%
Rat	BL Mouse	Omni Human	64.2%	bovine	65.6%
Rat	bovine	Omni Human	66.7%	mouse	62.0%

### Genes with single PAS V.S. genes with multi PAS

Some genes have more than one PAS while others have only one PAS. In this section, we build two new Rat PAS datasets, single PAS Rat datasets and multiple PAS Rat datasets, to investigate the performance of Poly(A)-DG on them. PolyA DB v3.2 [46] provides information about PAS and the gene they came from, we extract sequences with AATAAA PAS motif from genome database and divide them into two groups, gene have multiple PAS and gene have only one PAS. We train Poly(A)-DG on standard cross-species datasets and directly test on single and multiple PAS datasets. Table 9 shows the results of experiments. Predicting PAS in single PAS genes, Poly(A)-DG can achieve a much higher accuracy than it test on multiple PAS genes.

**Table 9:** Gene with single PAS V.S. gene with multiple PAS; target domain: Rat.

Source Domain		Single	Multiple
Omni Human	BL Mouse	**78.3%**	66.5%
Omni Human	bovine	**84.0%**	71.2%
BL Mouse	bovine	**78.5%**	67.2%

### Visualization of captured features in convolution filters

Poly(A)-DG directly processes DNA sequences and automatically extracts features instead of processing hand-crafted features. As discussed in the section CNN-MLP for feature extraction, convolution neural network in Poly(A)-DG is responsible for extracting motif features. In this section, we visualize the motif features that the convolution filters most likely to capture. Each convolution kernel in convolution layer has a sliding window with a width of 10 nucleotides to scan every 10-mer sub-sequences of input sequences. We utilize the values calculated by convolution kernels to visualize the learned motif features. For each convolution kernels, we search the 10-nt sub-sequence that kernels give the largest activation value for each DNA sequences sample. For all input DNA sequences, we construct position weight matrices and use WebLogo 3 [49] to visualize the pattern in sequence logo for each filters. We train Poly(A)-DG on cross-species datasets and send DNA sequences from target species to investigate the importance of motif in cross-species PAS identification.

**Fig 5:**
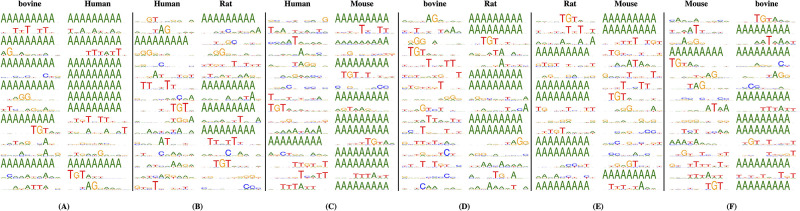
Visualization of convolution filters of Poly(A)-DG. (A)Source domain: BL Mouse and Rat. (B)Source domain: BL Mouse and bovine (C)Source domain: Rat and bovine. (D)Source domain: Omni Human and BL Mouse. (E)Source domain: Omni Human and bovine. (F)Source domain: Omni Human and Rat.

Fig 5 shows the entropy of motifs at each position that Poly(A)-DG captured for each of convolution filters. Given the same target species with different source domains, the Poly(A)-DG tends to capture different motifs. In general, we observe that most patterns that the convolution filters looking for across species are A-rich, T-rich and GT-rich DNA subsequences. These visualization outcomes confirmed the view proposed by previous studies that polyadenylation is regulated by A-rich, U-rich and GU-rich RNA sequences. Specifically, the TGT, AG(G), and AAAAAAAAA motifs appears frequently in four species, and some T-rich motifs like TTATTT are also popular among species. Since the Poly(A)-DG is trained on cross-species datasets, the motif captured by convolution kernels for target species can be regarded as species-invariant features. Besides invariant features shared by four species, we also notice some invariant features for specific species. For example, Poly(A)-DG is more sensitive to ATA for BL Mouse than other species, CCC is more important in bovine, and AAGA is more important in Human.

### Visualization of region impacts of DNA sequences

**Fig 6:**
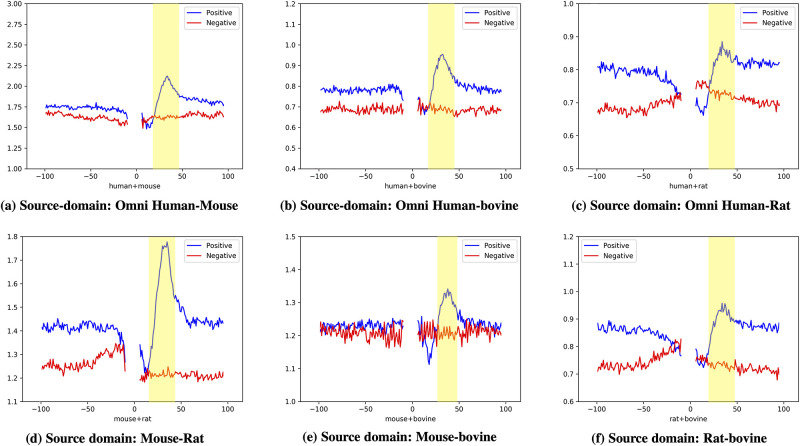
Visualization of position importance in PAS identification. (a)Source domain: Omni Human and BL Mouse. (b)Source domain: Omni Human and BL Mouse. (c)Source domain: Omni Human and BL Mouse. (d)Source domain: Omni Human and BL Mouse. (e)Source domain: Omni Human and BL Mouse. (f)Source domain: Omni Human and BL Mouse.

Different positions in PAS DNA sequences may contribute differently in PAS identification. To find out the part with the biggest difference between true and pseudo PAS sequences, we propose to visualize the contribution of every position in PAS recognition. As we mentioned in the previous subsection, Visualization of captured features in convolution filters, a filter in convolution layers of Poly(A)-DG scans every 10-mer sub-sequences with a stride of one nucleotide and it calculates a score to each scanned sub-sequence at every position. For 16 filters in convolution layers, we average the scores assigned by them at every position and obtain scores for every position when sequences are sent to Poly(A)-DG. For every two-species source domain, we first feed positive PAS sequences to the Poly(A)-DG to get positive position scores and then send negative PAS sequences to obtain negative scores. We plot positive and negative position values in Fig 6. The x-axis is the position of nucleotides in DNA samples, and the position of PAS ranges from 0 to 5. [-100, -1] is the upstream area of PAS and [6, 100] is downstream regions of PAS. The y-axis represents the importance of each position of the positive and negative samples in the feature extraction of the convolution kernel. The position values represent how sensitive the model is on the information of this position. If the position values are high, the information in this position is more important in PAS identification. The position with a larger position value difference between positive and negative sequences indicates that it is more significant in distinguishing true PAS from false ones. These six sub-figures show that the downstream regions of PAS contain more information than upstream ones, specifically, the regions located at 20-40 nt of downstream of PAS have the biggest differences between positive and negative position values. As proved by previous studies [6, 7], these regions with significant differences are usually the region of Poly(A) site where the cleavage operation take place. Due to transcription process goes from 5’ to 3’, the impact of the nucleotides contents might be highly unbalanced. However, from Fig 6, we observe the mean position value of upstream and downstream of poly(A) site is about the same. Besides the PAS and poly(A) site regions, we also observe that the position value of positive samples are generally higher than negative samples. The reason for this phenomenon is that Poly(A)-DG tends to look for T-rich or GT-rich motifs (discussed in section Visualization of captured features in convolution filters), and previous study [30, 50] confirmed that the upstream of PAS is T-rich and the downstream of poly(A) site is T or GT-rich. Therefore, we conjecture that the poly(A) site is the most essential elements in PAS identification.

### Predict the number of PAS on whole genome from chromosome 1 of rat

To further investigate the availability of Poly(A)-DG, we examine the performance of Poly(A)-DG on the whole genome of chromosome 1 of rat. PolyA-DB v3.2 provides 5,755 annotated PAS for chromosome 1 of rat. We first extract the whole genome of chromosome 1 in rat from genome database, and divided them into 206 nucleotides subsequences with a stride of 50 nucleotides. Owing to the PAS is a 6-nt motif, the same PAS contained by several consecutive 206-nt subsequences, though the position of PAS varies. In practical usage, the position of PAS within the DNA segments is unknown if we don’t have any annotation. Therefore, we label the sequences contain the entire PAS motif as positive samples and the remaining sequences as negative. Owing to the different strands of PAS, we reverse the chromosome 1 of rat and repeat the process mentioned above to extract positive and negative PAS sequences. The final established rat chromosome 1 PAS datasets contains 11,541,504 negative PAS sequences and 17,920 DNA sequences that contains PAS. We predict the rat chromosome 1 PAS datasets by Poly(A)-DG trained on three cross-species source domains. We report the false positive rate (FPR), false negative rate (FNR), and accuracy in Table 10. The FPR is about 25%, FNR is about 65% and the accuracy is around 75%. We notice that the high FNR is caused by the subsequence dividing strategy. Poly(A)-DG trains on our standard PAS datasets which the location of PAS is in the middle of sample for all positive DNA sequences. However in the rat chromosome 1 PAS dataset, due to the subsequence dividing strategy mentioned above, the position of PAS is random. The position shift of PAS may greatly affect the performance of Poly(A)-DG to identify the positive sequences. Our experiments indicates that Poly(A)-DG is promising in detecting PAS from whole genome as an aid tool to help researchers.

**Table 10:** Predict PAS on chromosome 1 of rat; FPR: False positive rate; FNR: False negative rate.

Source Domain		FPR	FNR	Acc.
Omni Human	BL Mouse	24.91%	66.36%	75.02%
Omni Human	bovine	30.76%	58.52%	69.15%
BL Mouse	bovine	22.22%	69.52%	77.70%

### Homology effect of PAS dataset

We sought to examine whether the homology sequence among four PAS datasets affect the PAS identification. According to previous works [34, 51], we adopt the CD-HIT [52] to identify pairs of homology sequence among Omni Human, BL Mouse, Rat and bovine PAS datasets with the lowest threshold 0.8. We filtered detected pairs of homology sequence and the number of PAS sequence in Omni Human is reduced from to 33,575, the number of PAS sequence in BL Mouse is reduced from to 34,714, the samples of Rat is reduced from to 28,057, and the samples of bovine PAS sequence is reduced from to 20,272. We retrained Poly(A)-DG on filtered cross-species PAS datasets and evaluate it on filtered target species. Empirical results in Table 11 show that the performance of Poly(A)-DG will not be obviously influenced by homology pairs of DNA sequences, and in some indices, the accuracy of filtered datasets is higher than that on standard datasets.

**Table 11:** Performance of Poly(A)-DG on standard cross-species PAS dataset and filtered cross-species PAS dataset.

Source Domain		Target	filtered	standard	Target	filtered	standard
Omni Human	BL Mouse	Rat%	**68.8**%	68.5%	bovine	**75.5**%	75.3%
Omni Human	bovine	BL Mouse	**71.0**%	68.9%	Rat	68.3%	**69.4**%
BL Mouse	bovine	Omni Human	74.4%	**75.0**%	Rat	66.7%	**67.6**%
Rat	bovine	Omni Human	**74.8**%	73.8%	BL Mouse	**70.3**%	67.9%
Rat	BL Mouse	Omni Human%	70.1%	**72.3**%	bovine	70.9%	**72.4**%
Omni Human	Rat	BL Mouse	**71.3**%	69.7%	bovine	75.8%	**76.0**%

### Conclusion

In our work, we propose a neural-network-based domain generalization model which can be trained on cross-species PAS datasets and directly applied to identify PAS from a new species. In our experiments, we mix each two of four datasets, Omni Human, BL Mouse, Rat, and bovine to build cross-species training sets and evaluate our method on rest species. According to the experimental results, Poly(A)-DG achieves relatively high accuracy over balanced cross-species datasets. Moreover, we investigate the performance of Poly(A)-DG on insufficient and imbalanced cross-species training sets. We observe that the performance of Poly(A)-DG would not decline drastically when the available training data significantly decreases. On imbalanced cross-species training sets, Poly(A)-DG works relatively stably when the size of one species varies. on imbalanced PAS positive-negative ratio cross-species datasets, the Poly(A)-DG can still work and detect the PAS even the number of negative samples is five times larger than the positive ones in the training domains. We conjecture that Poly(A)-DG outperforms existing methods may owe to its ability to learn invariant distributions between different species. Experimental results show that Poly(A)-DG, which is well adaptable to different species, provides us a promising way to address practical PAS identification problems. We investigate the homology effect of PAS datasets and empirical results indicate that Poly(A)-DG is slightly affected by the homology DNA sequences. We examine the performance of Poly(A)-DG in identifying the PAS from genes with single poly(A) site and multiple poly(A) site, and find that our model works better on gene with only one poly(A) site. We try to apply Poly(A)-DG on the whole genome from chromosome 1 of rat to evaluate the availability of Poly(A)-DG on practical usage and the relatively low false positive rate means that Poly(A)-DG may be a tool to help researchers to exclude DNA sequences that don’t contain PAS. We visualize the convolution filters and find most captured motifs are A-rich, T-rich and GT-rich DNA subsequences. We also visualize the position impact of PAS identification and find the 20-40 nt downstream of PAS contribute most in distinguishing true PAS from pseudo ones. We understand the RNA binding proteins (RBPs) are essential in polyadenylation regulation, while the current version of Poly(A)-DG doesn’t support the RBPs motif matching and the RBPs variants discovering among species. We will further work on this issue and hope to facilitate it in future works.

## Supporting information

S1 Supporting Information Supplementary materials. (PDF)

S1 File Human bovine and mouse Poly(A) signal datasets. These three datasets are produced by previous researchers and we use them in our experiments.
(ZIP)

S2 File Rat true Poly(A) signal datasets. We extract labeled Poly(A) signals sequences following the PolyA DB v3.2 dataset [46].
(TXT)

S3 File Rat pseudo Poly(A) signal datasets. The pseudo Poly(A) signal datasets used in experiments.
(ZIP)
