# Supplementary material for: Poly(A)-DG: A deep-learning-based domain generalization method to identify cross-species Poly(A) signal without prior knowledge from target species
Source: PLoS Comput Biol. 2020 Nov 5;16(11):e1008297. doi: 10.1371/journal.pcbi.1008297 (PMC7671507; doi:10.1371/journal.pcbi.1008297)
Supplement: S1 Supporting Information — (PDF) [file pcbi.1008297.s001.pdf]

Supplemental Materials for Poly(A)-DG: a deep-learning-based domain generalization method to identify cross-species Ploy(A) Signal without prior knowledge

Yumin Zheng[1], Haohan Wang[2], Yang Zhang[3], Xin Gao[4], Eric P. Xing[5], and Min Xu[3,]*
[1]*International School, Beijing University of Posts and Telecommunications, Beijing, China*
[2]*Language Technologies Institute, School of Computer Science, Carnegie Mellon University, Pittsburgh, PA, USA*
[3]*Computational Biology Department, School of Computer Science, Carnegie Mellon University, Pittsburgh, PA, USA*
[4] *Computational Bioscience Research Center (CBRC), Computer, Electrical and Mathematical Sciences and Engineering (CEMSE) Division, King Abdullah University of Science and Technology, Thuwal, Saudi Arabia*
[5]*Machine Learning Department, School of Computer Science, Carnegie Mellon University, Pittsburgh, PA, USA*

1 Architecture & Details of Implementation

Tensorflow implementation of Poly(A)-DG can be found on the GitHub.

1.1 MLP

The inputs of MLP are shuffled and one-hot encoded DNA sequences. The hidden layers of MLP have 128 nodes and we use a ReLU to activate the outputs. The architecture of our MLP module is shown in Figure [1].

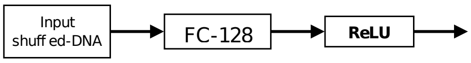

Figure 1: Flow chart of MLP

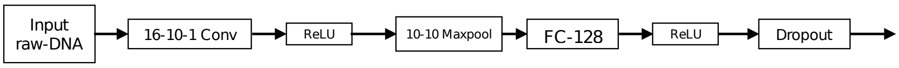

Figure 2: Flow chart of CNN

1.2 CNN

The inputs of MLP are one-hot encoded DNA sequences. We apply 16 1-D convolution kernels, which size is 10*1 and stride is 1, in the convolution layer and use ReLU to activate the feature maps. After ReLU, we place a 1-D Max-Pooling layer with a 10*1 kernel and stride of 10. Then we flatten the output of the max pooling layer and fed the feature maps to a fully-connected layer with 128 hidden nodes and use ReLU to activate its output. The Dropout technique is applied to alleviate over-fitting. The architecture of our CNN module is shown in Figure [2]

1.3 Domain generalization & Prediction

We concatenate representations extracted by MLP and CNN and feed them into a fully-connected layer with 2 hidden nodes. Then we use the HEX block to process concatenated features and a softmax classifier to output the prediction of samples. The operation of HEX and classifier is shown in Figure [3]

1.4 Hyperparameters

The batch size of our experiments is 256. Following [1], we use hyperparameters searching method to find the most appropriate hyperparameters for our experiments. To sample a real number in the interval [a, b] with log-uniform, we first uniformly sample a real number $x \in [0, 1]$, then return $10^{(log_{10}b - log_{10}a)x + log_{10}a}$. The search range and sampling method of each hyper-parameter can are presented in Table [1].

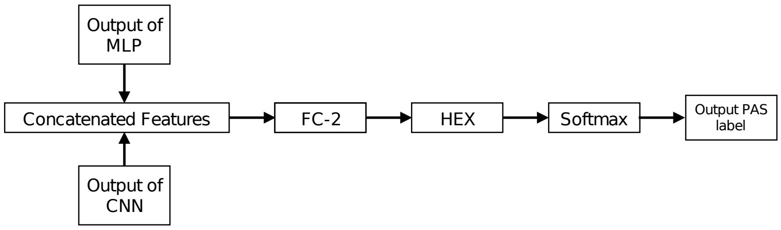

Figure 3: Flow chart of HEX and classifier

Parameter	Search Range	Sampling Method
Learning Rate	$[1e^{-1}, 1e^{-3}]$	log-uniform
Keep Probability	{0.2, 0.5, 0.8}	Random choice

Table 1: Search range and sampling method for hyper-parameters

2 Shuffled V.S. Raw DNA sequences in MLP

We follow the same strategy as Wang et al. [2] to evaluate the ability of MLP to identify PAS. In this section, we mix BL Mouse, Omni Human and Rat to build a cross-species PAS data set. We compared the performance of MLP when the inputs are Shuffled DNA sequences or Raw DNA sequences. For Shuffled DNA sequences, we shuffle the internal order of each DNA sequence. The parameters are trained to minimize the prediction risk of PAS instead of species. We extracted the representations of MLP for different inputs and used these representations as features to test the five-fold cross-validated Naïve Bayes classifiers accuracy of predicting PAS. With two choices of learning rates, we repeated this for every epoch through 100 epochs of training and reported the mean and standard deviation over 100 epochs in Table [2]: After shuffling, MLP is less likely to recognize PAS in cross-species data sets.

	Random	Raw(1e-5)	Shuffled(1e-5)	Raw(1e-3)	Shuffled(1e-3)
PAS	0.5	0.643±0.030	0.522±0.002	0.587±0.001	0.523±0.001

Table 2: Accuracy of PAS classification

3 Establish Rat PAS data set

3.1 Build positive data set

We use rat's PolyA annotations from PolyA_DB v3.2 to build a positive rat PAS data set. In PolyA_DB v3.2, which is an annotated Poly(A) sites data set mainly based on NCBI Gene and RefSeq databases, the Poly(A) signal is located within 40-nt upstream from the Poly(A) site. We choose samples that contain Poly(A) sites from rat samples in PolyA_DB v3.2. Then we down load the rat genome sequence database (in fasta format) from the UCSC Genome Browser and use the get fasta function from the bedtools to extract the corresponding DNA sequences in the rat genome. We select 11 patterns of Poly(A) signal in this database including AATAAA, AGTAAA, TATAAA, CATAAA, GATAAA, AATATA, AATACA, AATAGA, AAAAAG, ACTAAA and ATTAAA. We scan the 40-nt upstream from the Poly(A) site in each sequence to find the poly(A) signal of it. (Some sequences have more than one potential Poly(A) signal motifs, we randomly select one as Poly(A) signal) Then we crop the 200 nt genomic sequences flanking the Poly(A) signal as one sample in our Poly(A) signal data set. (100-nt upstream and 100-nt downstream

from the Poly(A) signal) Last, we classify positive Poly(A) signal sequences into different groups by their Poly(A) signal patterns.

3.2 Build pseudo data set

According to the Rat BodyMap database, we select genes which are expressed in the rat's brain. We only select those gene segments which are not annotated to contain PAS to make sure the reliability of our pseudo-data set. Then we use the get fasta function to extract the selected DNA sequences and search motifs that are the same as PAS patterns. When we find the motifs the same as PAS patterns, we crop 200 nt genomic sequences, 100-nt upstream and 100-nt downstream, flanking the pseudo-motif as one pseudo sample. The number of pseudo PAS sequences is the same as the number of true sequences and the number of true PAS sequences from each PAS motif is the same as the number of pseudo ones as well.

4 Experimental results of training with limited data

We train Poly(A)-DG on insufficient training data to evaluate the performance of Poly(A)-DG when the source domain is small. We use six cross-species datasets in the evaluation, Human-Mouse, Human-Rat, Human-bovine, Mouse-Rat, Mouse-bovine and Rat-bovine. The experimental results are shown in Figure 4.

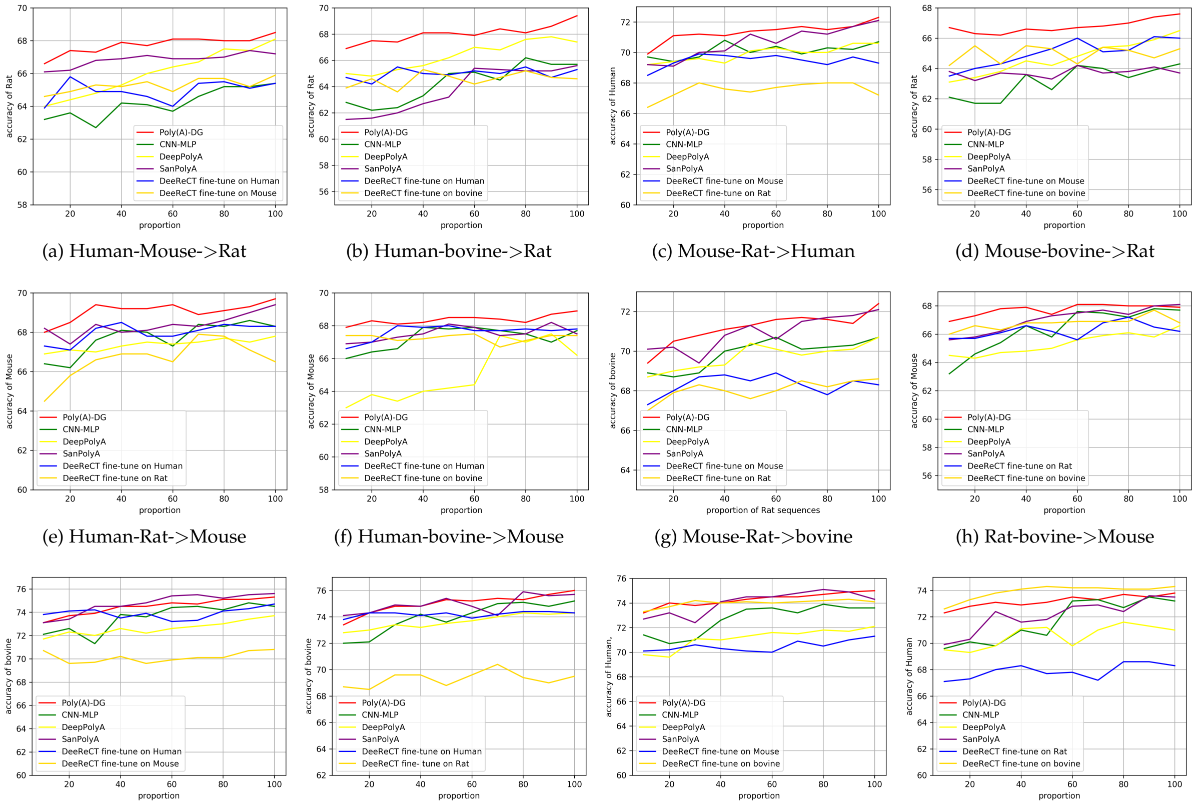

(a) Human-Mouse->Rat (b) Human-bovine->Rat (c) Mouse-Rat->Human (d) Mouse-bovine->Rat

(e) Human-Rat->Mouse (f) Human-bovine->Mouse (g) Mouse-Rat->bovine (h) Rat-bovine->Mouse

(i) Human-Mouse->bovine (j) Human-Rat->bovine (k) Mouse-bovine->Human (l) Rat-bovine->Human

Figure 4: Experimental results of training with limited data

5 Experimental results of training with imbalanced species

To investigate the performance of Poly(A)-DG on imbalanced species source domains, we conduct experiments by fix the DNA sequences number of one species in the source domain, and control the number of DNA sequences from the other species. We have six cross-species source domain, Human-Mouse, Human-Rat, Human-bovine, Mouse-Rat, Mouse-bovine and Rat-bovine. We use the name of species to label each source domain, and the first species in the name as first species for the source domain. For example, the Human-Mouse dataset, the first species is human and the second species is mouse. We show the experimental results in Figure 5 and Figure 6. The Figure 5 shows the experiments conducted on datasets with the DNA sequences number of first species is fixed and we plot the empirical results of experiments on source domains with the sample number of second species is fixed in the Figure 6.

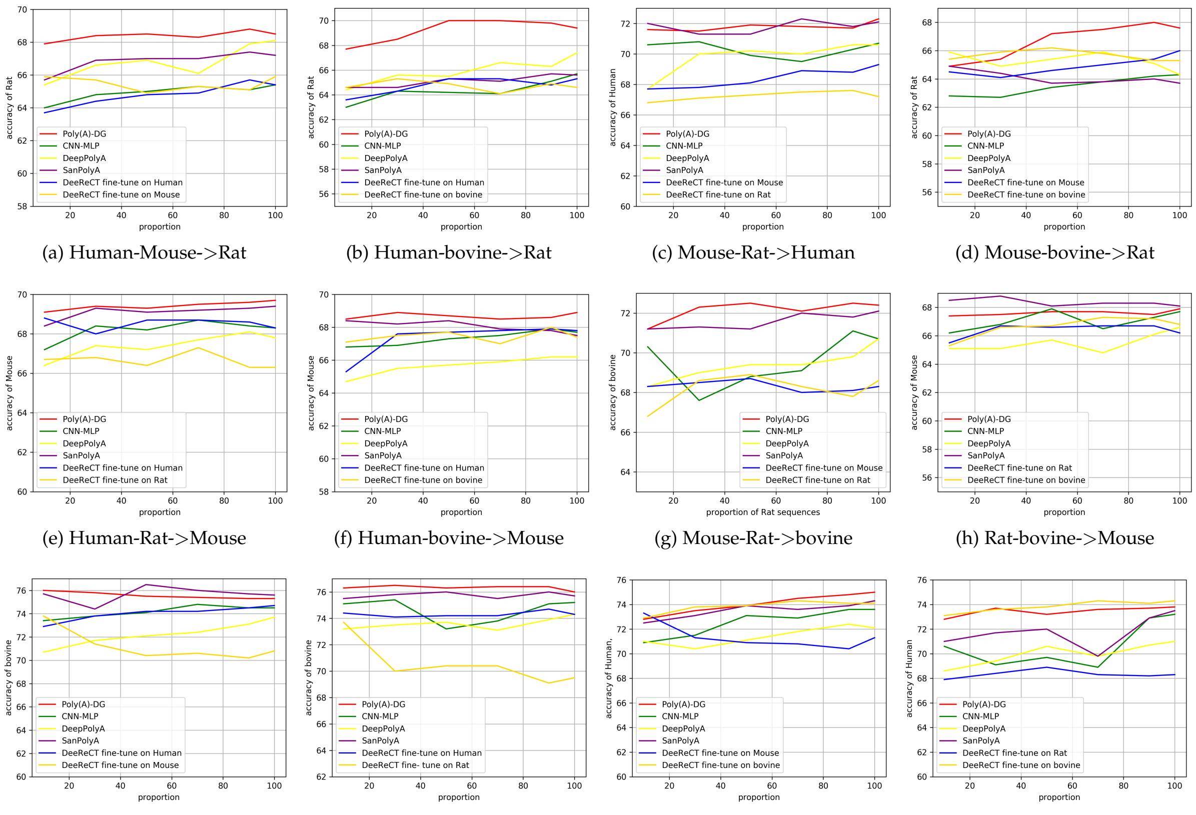

(a) Human-Mouse->Rat (b) Human-bovine->Rat (c) Mouse-Rat->Human (d) Mouse-bovine->Rat

(e) Human-Rat->Mouse (f) Human-bovine->Mouse (g) Mouse-Rat->bovine (h) Rat-bovine->Mouse

(i) Human-Mouse->bovine (j) Human-Rat->bovine (k) Mouse-bovine->Human (l) Rat-bovine->Human

Figure 5: Experimental results of training with imbalanced species, the number of samples in the first species in source domain is fixed

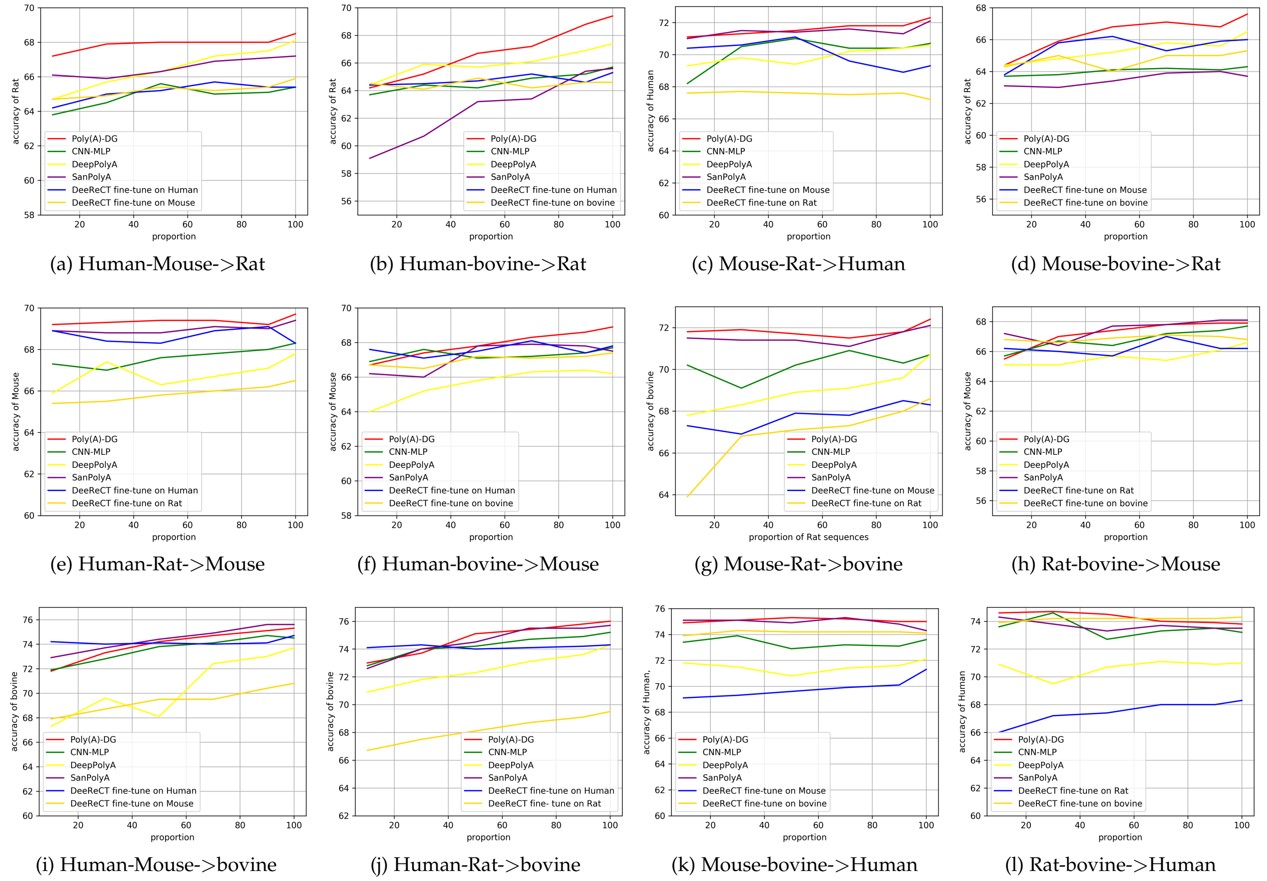

(a) Human-Mouse->Rat (b) Human-bovine->Rat (c) Mouse-Rat->Human (d) Mouse-bovine->Rat

(e) Human-Rat->Mouse (f) Human-bovine->Mouse (g) Mouse-Rat->bovine (h) Rat-bovine->Mouse

(i) Human-Mouse->bovine (j) Human-Rat->bovine (k) Mouse-bovine->Human (l) Rat-bovine->Human

Figure 6: Experimental results of training with imbalanced species, the number of samples in the second species in source domain is fixed

6 Experimental results of training with imbalanced PAS positive-negative ratio

We show the experimental results of Poly(A)-DG training on source domains that the number of positive and negative samples are 1:10. In this scenario, the number of positive samples in the source domain is about 2000-4000, while the number of negative samples ranges from 20,000 to 40,000. The ratio of the number of positive and negative samples in the target domain is 1:1. We show the experimental results in Table 3. The accuracy of Poly(A)-DG in many source domains is close to or equals to the random (50%), in other source domains, Poly(A)-DG works with a relatively low accuracy.

Table 3: **Imbalanced PAS positive-negative ratio is 1:10 for the source domain**

Source Domain		Target	Accuracy	Target	Accuracy
Omni Human	BL Mouse	Rat	52.0%	bovine	53.2%
Omni Human	Rat	BL Mouse	55.7%	bovine	57.9%
Omni Human	bovine	BL Mouse	56.0%	Rat	56.7%
bovine	BL Mouse	Omni Human	52.5%	Rat	50.0%
Rat	BL Mouse	Omni Human	52.8%	bovine	52.8%
Rat	bovine	Omni Human	55.4%	mouse	54.6%

References

[1] Xia, Z. and Li, Y. and Zhang, B. and Li, Z. and Hu, Y. and Chen, W. and Gao, X.(2018)DeeReCT-PolyA: a robust and generic deep learning method for PAS identification, *Bioinformatics.*

[2] Wang, H. and He, Z. and Lipton, Z. C. and Xing, E. P.(2019)Learning Robust Representations by Projecting Superficial Statistics Out, *International Conference on Learning Representations*
